# Interactions between Activation and Repolarization Restitution Properties in the Intact Human Heart: In-Vivo Whole-Heart Data and Mathematical Description

**DOI:** 10.1371/journal.pone.0161765

**Published:** 2016-09-02

**Authors:** Michele Orini, Peter Taggart, Neil Srinivasan, Martin Hayward, Pier D. Lambiase

**Affiliations:** 1 Institute of Cardiovascular Science, University College London, London, United Kingdom; 2 Barts Heart Centre, St Bartholomews Hospital, London, United Kingdom; 3 The Heart Hospital, University College London Hospitals, London, United Kingdom; Universiteit Gent, BELGIUM

## Abstract

**Background:**

The restitution of the action potential duration (APDR) and conduction velocity (CVR) are mechanisms whereby cardiac excitation and repolarization adapt to changes in heart rate. They modulate the vulnerability to dangerous arrhythmia, but the mechanistic link between restitution and arrhythmogenesis remains only partially understood.

**Methods:**

This paper provides an experimental and theoretical study of repolarization and excitation restitution properties and their interactions in the intact human epicardium. The interdependence between excitation and repolarization dynamic is studied in 8 patients (14 restitution protocols, 1722 restitution curves) undergoing global epicardial mapping with multi-electrode socks before open heart surgery. A mathematical description of the contribution of both repolarization and conduction dynamics to the steepness of the APDR slope is proposed.

**Results:**

This study demonstrates that the APDR slope is a function of both activation and repolarization dynamics. At short cycle length, conduction delay significantly increases the APDR slope by interacting with the diastolic interval. As predicted by the proposed mathematical formulation, the APDR slope was more sensitive to activation time prolongation than to the simultaneous shortening of repolarization time. A steep APDR slope was frequently identified, with 61% of all cardiac sites exhibiting an APDR slope > 1, suggesting that a slope > 1 may not necessarily promote electrical instability in the human epicardium. APDR slope did not change for different activation or repolarization times, and it was not a function of local baseline APD. However, it was affected by the spatial organization of electrical excitation, suggesting that in tissue APDR is not a unique function of local electrophysiological properties. Spatial heterogeneity in both activation and repolarization restitution contributed to the increase in the modulated dispersion of repolarization, which for short cycle length was as high as 250 ms. Heterogeneity in conduction velocity restitution can translate into both activation and repolarization dispersion and increase cardiac instability. The proposed mathematical formulation shows an excellent agreement with the experimental data (correlation coefficient *r* = 0.94) and provides a useful tool for the understanding of the complex interactions between activation and repolarization restitution properties as well as between their measurements.

## Introduction

Cardiac restitution refers to mechanisms whereby the electrical excitation and recovery adapt to heart rate changes. At fast heart rates, recovery from electrical excitation becomes faster while conduction velocity simultaneously decreases [1, 2]. The adaptation of repolarization and conduction velocity with respect to heart rate is described by the restitution curve, and it has been shown to modulate the propensity to dangerous ventricular arrhythmia [3–5]. However, the precise mechanisms linking restitution properties to arrhythmia are only partially understood.

The action potential duration restitution (APDR) curve represents the action potential duration as a function of the preceding diastolic interval or local cycle length (CL) [2]. It is usually almost flat for CL longer than 550 ms and steeper for shorter CL. Several studies have shown that the steepness of the APDR curve is relevant to arrhythmogenesis [4, 6–8].

Conduction velocity also displays restitution properties and it decreases at short CL [9]. Conversely, activation time increases at short CL, and activation time restitution (ATR) is often used as a surrogate for CVR [10, 11]. Steep and broad CVR and ATR curves have been shown to interact with APDR and promote spatially discordant alternans [12–14], a potent arrhythmogenic substrate [15, 16].

Several studies have focused on both APD and conduction velocity restitutions in computational [7, 12, 13, 17–22] and animal [9, 14, 16, 23] models, but data describing the interactions between repolarization and conduction dynamics in the in-vivo human cardiac tissue is scant. Although APDR and CVR are often treated as independent phenomena, they occur simultaneously in tissue and a tight mutual interaction exists between them and their measurements. The slope of the APDR curve is determined by changes in both the action potential duration and diastolic interval. Since the latter depends on activation time, APDR is strongly affected by conduction dynamics.

The interdependence between measurements of APDR and conduction kinetics are crucial to provide a correct interpretation of the mechanistic link between restitution and arrhythmogenesis and they have not been investigated exhaustively in the intact human heart. This paper provides the results of a theoretical and experimental investigation of the APDR slope and its main determinants, i.e. electrical excitation and recovery dynamics. Data collected during whole-heart epicardial mapping of the intact human heart is presented, and a mathematical description of the dynamic interactions between the main cardiac intervals during heart rate changes is proposed. Overall, this study aims at improving our understanding of the restitution properties and their link to arrhythmogenesis.

## Mathematical description of restitution properties

### Interactions between cardiac intervals

**Fig 1:**
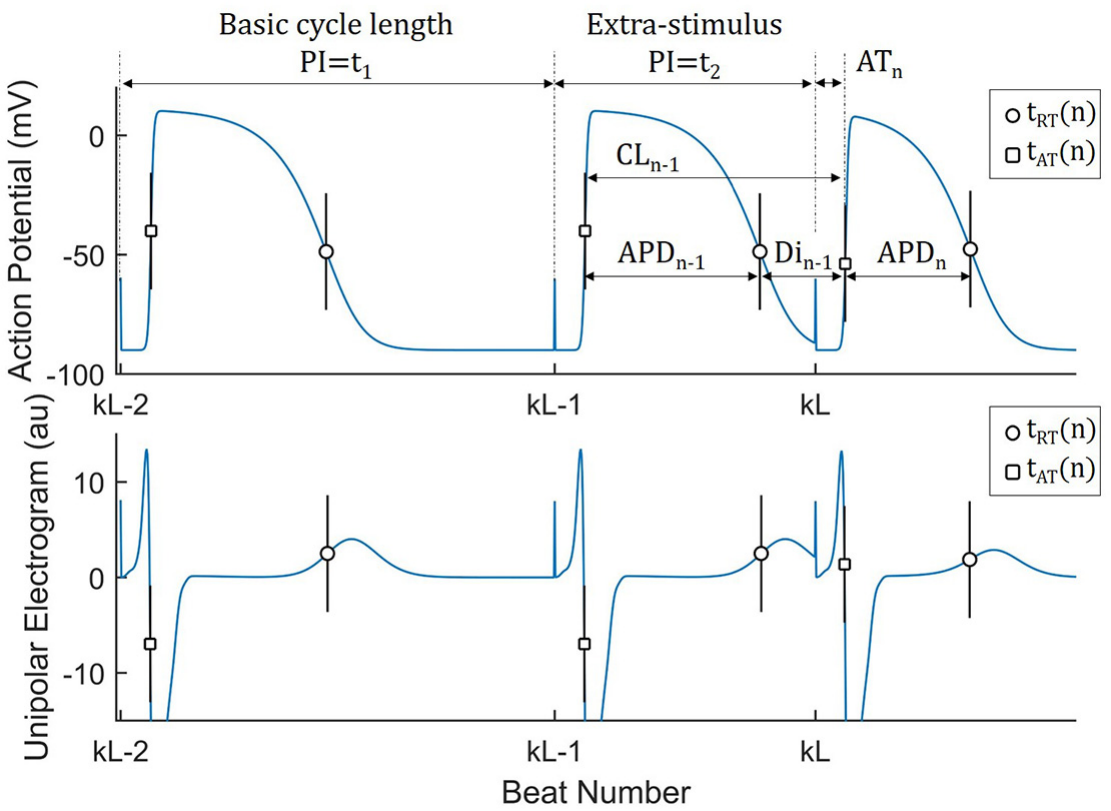
Main cardiac intervals from action potentials (AP) and unipolar electrograms recording at a given cardiac site *x* during an S1–S2 restitution protocol (schematic). Sub-index *x* is omitted for clarity. PI: Pacing interval; AT: Activation time, from pacing stimulus to AP upstroke $t_{n}^{\text{AT}}$; RT: Repolarization time, form pacing stimulus to AP recovery $t_{n}^{\text{RT}}$. APD: Action potential duration, $APD\left(n\right)=RT\left(n\right)-AT\left(n\right)=t_{n}^{\text{RT}}-t_{n}^{\text{AT}}$; DI: Diastolic interval, form AP recovery to following AP upstroke, $DI_{n}=t_{n}^{\text{AT}}-t_{n-1}^{\text{RT}}$. CL: Local cycle length, from AP upstroke to following AP upstroke, $CL\left(n\right)=t_{n}^{\text{AT}}-t_{n-1}^{\text{AT}}$. The APD restitution curve represents *APD*_*n*_ as a function of *DI*_*n*−1_ for different S1–S2 PI, *t*_2_. See Eqs (1)–(5) for a description of their interactions.

Local activation (AT) and repolarization times (RT) mark the onset and the recovery of electrical excitation, and they are usually measured from a common reference time:

$$\begin{array}{c} AT_{x,n}=t_{x,n}^{\text{AT}}-t_{n}^{0} \end{array}$$ (1)

$$\begin{array}{c} RT_{x,n}=t_{x,n}^{\text{RT}}-t_{n}^{0} \end{array}$$ (2)

where *n* is the heart beat number, *x* indicates the position of a given cardiac site, while $t_{n}^{0}$, $t_{x,n}^{\text{AT}}$ and $t_{x,n}^{\text{RT}}$ are the time of occurrence of the triggering event used as temporal reference (e.g. a pacing stimulus), and local activation and repolarization, respectively (Fig 1). The action potential duration (APD) is the duration of the local electrical excitation and represents the electrical systole. The electrical diastole is represented by the diastolic interval (DI), which goes from RT to the following AT. Fig 1 provides a diagrammatic representation of all main cardiac intervals from action potentials and unipolar electrograms. Considering two consecutive cardiac cycles, these intervals are related by the following expressions:

$$\begin{array}{c} CL_{x,n}=t_{x,n}^{\text{AT}}-t_{x,n-1}^{\text{AT}}=PI_{n}+AT_{x,n}-AT_{x,n-1} \end{array}$$ (3)

$$\begin{array}{c} APD_{x,n}=RT_{x,n}-AT_{x,n} \end{array}$$ (4)

$$\begin{array}{c} DI_{x,n-1}=\underset{CL_{x,n-1}}{\underset{︸}{PI_{n-1}+AT_{x,n}-AT_{x,n-1}}}-APD_{x,n-1} \end{array}$$ (5)

where *CL*_*x*,*n*_ is the local cycle length, and $PI_{n}=t_{n}^{0}-t_{n-1}^{0}$ is the pacing interval, defined as the interval between two consecutive pacing stimuli. Note that in tissue, contrary to what happens in an isolated cell, local CL and PI can differ because of local AT and conduction velocity beat-to-beat variability. That is, if *AT*_*x*,*n*_ ≠ *AT*_*x*,*n*−1_ then *PI*_*n*_ ≠ *CL*_*x*,*n*_. Therefore, within a given beat, PI is equal to the local CL only if CVR is not engaged.

An S1–S2 restitution protocol in an established procedure to explore the relationship between the APD and its preceding DI over a range of different heart rates [2]. After a train of electrical stimuli is delivered to pace the heart at a constant pacing interval (the basic cycle length *t*_1_), an extra-stimulus is delivered and the APD during the post extra-stimulus beat, i.e. *APD*_*x*,*n*_, is registered and studied as a function of the preceding DI, i.e. *DI*_*x*,*n*−1_ (see Fig 1). A general formulation for the APD registered after the extra-stimulus S2 and its preceding DI can be written as:

$$\begin{array}{c} APD_{x,n}\left(t_{1},t_{2}\right)=RT_{x,n}\left(t_{1},t_{2}\right)-AT_{x,n}\left(t_{1},t_{2}\right) \end{array}$$ (6)

$$\begin{array}{c} DI_{x,n-1}\left(t_{1},t_{2}\right)=t_{2}+AT_{x,n}\left(t_{1},t_{2}\right)-AT_{x,n-1}\left(t_{1}\right)-APD_{x,n-1}\left(t_{1}\right) \end{array}$$ (7)

where *t*_1_ and *t*_2_ are variables that can take real positive values higher than the effective refractory period of the tissue and all terms related to beat *n* − 1 do not depend on *t*_2_ because they occur before the extra-stimulus is delivered. Note that this expression does not include the effect of intrinsic [24–26] or random [27] fluctuations of cardiac intervals which may be unrelated to restitution properties.

### The APD restitution curve

In the following, the sub-index *x* will be omitted and the sub-index *n* will represent the post extra-stimulus beat. Furthermore, it is assumed that the basic cycle length is fixed at a constant value *t*_1_. As a result, the steepness of the APDR curve, defined as the derivative of the APD registered after the extra-stimulus with respect to its preceding DI, is a function of the S1–S2 coupling interval *t*_2_:

$$\begin{array}{c} \alpha \left(DI\right)=\frac{dAPD_{n}\left(t_{2}\right)}{dDI_{n-1}\left(t_{2}\right)}=\frac{dAPD_{n}\left(t_{2}\right)}{dt_{2}}{\left(\frac{dDI_{n-1}\left(t_{2}\right)}{dt_{2}}\right)}^{-1} \end{array}$$ (8)

and variations of APD and DI with respect to *t*_2_ are:

$$\begin{array}{c} \frac{dAPD_{n}\left(t_{2}\right)}{dt_{2}}=APD_{n}^{\prime }\left(t_{2}\right)=RT_{n}^{\prime }\left(t_{2}\right)-AT_{n}^{\prime }\left(t_{2}\right) \end{array}$$ (9)

$$\begin{array}{c} \frac{dDI_{n-1}\left(t_{2}\right)}{dt_{2}}=DI_{n-1}^{\prime }\left(t_{2}\right)=1+AT_{n}^{\prime }\left(t_{2}\right) \end{array}$$ (10)

Therefore, Eq (8) becomes:

$$\begin{array}{c} \alpha \left(DI\right)=\frac{RT_{n}^{\prime }\left(t_{2}\right)-AT_{n}^{\prime }\left(t_{2}\right)}{1+AT_{n}^{\prime }\left(t_{2}\right)} \end{array}$$ (11)

A graphical interpretation of this mathematical expression is proposed in Fig A in S1 File. A preliminary formulation of this model was presented in [28]. This formulation shows that the steepness of the APDR curve depends on changes in AT, $AT_{n}^{\prime }\left(t_{2}\right)$, and consequently on CVR. This is an important, yet often overlooked, property of the APDR curve. In absence of CVR, as for example in single cell experiments, the steepness of the APDR curve Eq (11) becomes:

$$\begin{array}{c} AT_{n}^{\prime }\left(t_{2}\right)=0\;\Rightarrow \;\alpha \left(DI\right)=APD_{n}^{\prime }\left(t_{2}\right)=RT_{n}^{\prime }\left(t_{2}\right) \end{array}$$ (12)

Therefore $RT_{n}^{\prime }\left(t_{2}\right)$ can be considered as the steepness of the APDR curve in absence of CVR. When CVR is engaged, AT tends to progressively increase for shorter coupling intervals [9, 11, 13, 14, 23]. This implies that, for short *t*_2_, $AT_{n}^{\prime }\left(t_{2}\right)\leq 0$ and therefore $\alpha \left(DI\right)\geq APD_{n}^{\prime }\left(t_{2}\right)\geq RT_{n}^{\prime }\left(t_{2}\right)$. This demonstrates that whenever CVR is engaged, it contributes to make the APDR curve steeper. Furthermore, Eq (11) shows that $AT_{n}^{\prime }\left(t_{2}\right)$ is related to *α*(*DI*) by a non-linear hyperbolic function, meaning that small changes in $AT_{n}^{\prime }\left(t_{2}\right)$ can induce a dramatic increase in the APDR slope, with for instance *α*(*DI*)→+∞ for $AT_{n}^{\prime }\left(t_{2}\right)\to -1$.

The description of the APDR slope in Eq (11) is based on the assumption that the APD and DI only depend on *t*_2_. However, these conditions are often not verified because of intrinsic and random fluctuations [24–26], and inaccuracies in signal delineation.

#### Linear approximation for APDR slope calculation

In experimental studies, the APDR slope is often computed by linear fitting inside sliding windows that span a short range of DI [29]. Assuming that for a given range of coupling intervals *t*_2_, *RT*_*n*_(*t*_2_) and *AT*_*n*_(*t*_2_) can be described by linear functions such as:

$$\begin{array}{c} RT_{n}\left(t_{2}\right)=\alpha_{\text{RT}}^{0}\cdot t_{2}+c_{\text{RT}} \end{array}$$ (13)

$$\begin{array}{c} AT_{n}\left(t_{2}\right)=\alpha_{\text{AT}}^{0}\cdot t_{2}+c_{\text{AT}} \end{array}$$ (14)

then *α*(*DI*) becomes a constant value representing the APDR slope:

$$\begin{array}{c} \alpha =\frac{\alpha_{\text{APD}}^{0}}{1+\alpha_{\text{AT}}^{0}}=\frac{\alpha_{\text{RT}}^{0}-\alpha_{\text{AT}}^{0}}{1+\alpha_{\text{AT}}^{0}} \end{array}$$ (15)

In this expression, $\alpha_{\text{APD}}^{\text{0}}$, $\alpha_{\text{RT}}^{\text{0}}$ and $\alpha_{\text{AT}}^{\text{0}}$ are the slopes of the APD, RT and AT restitution curves evaluated with respect to the S1–S2 coupling interval *t*_2_. The sensitivity of the APD restitution slope, *α*, to changes in $\alpha_{\text{RT}}^{\text{0}}$ and $\alpha_{\text{AT}}^{\text{0}}$, can be quantified as:

$$\begin{array}{c} \frac{d\alpha }{d\alpha_{\text{RT}}^{\text{0}}}=\frac{d\alpha }{d\alpha_{\text{APD}}^{\text{0}}}=\frac{1}{1+\alpha_{\text{AT}}^{\text{0}}}\; \end{array}$$ (16)

$$\begin{array}{c} \frac{d\alpha }{d\alpha_{\text{AT}}^{\text{0}}}=-\frac{\alpha_{\text{RT}}^{\text{0}}+1}{{\left(1+\alpha_{\text{AT}}^{\text{0}}\right)}^{2}}=-(\frac{d\alpha }{d\alpha_{\text{RT}}^{\text{0}}}+\frac{\alpha_{\text{APD}}^{\text{0}}}{{\left(1+\alpha_{\text{AT}}^{\text{0}}\right)}^{2}}) \end{array}$$ (17)

Note that *α* increases whenever *RT*_*n*_(*t*_2_) and *AT*_*n*_(*t*_2_) become steeper, i.e. when $\alpha_{\text{RT}}^{\text{0}}$ becomes more positive and $\alpha_{\text{AT}}^{\text{0}}$ more negative. Also, since both $\alpha_{\text{RT}}^{\text{0}}$ and $\alpha_{\text{AT}}^{\text{0}}$ are usually ≥ −1, $d\alpha /d\alpha_{\text{RT}}^{\text{0}}\geq 0$ and $d\alpha /d\alpha_{\text{AT}}^{\text{0}}<0$. More importantly, $|d\alpha /d\alpha_{\text{AT}}^{\text{0}}|>|d\alpha /d\alpha_{\text{RT}}^{\text{0}}|$ if $\alpha_{\text{APD}}^{\text{0}}>0$, i.e. as long as APD decreases with the S1-S2 coupling interval (which is almost always verified), *α* is more sensitive to changes in AT, $\alpha_{\text{AT}}^{\text{0}}$, than to changes in RT, $\alpha_{\text{RT}}^{\text{0}}$.

### Conduction velocity restitution

Given Eqs (4) and (5), an interaction between APDR and CVR must exist, because both the APD and the DI depends on AT. In a cable, AT and CV are related by:

$$\begin{array}{c} AT_{n}\left(t_{2}\right)=\frac{r}{v(t_{2})};\;AT_{n}^{\prime }\left(t_{2}\right)=-\frac{v^{\prime }\left(t_{2}\right)}{v^{2}\left(t_{2}\right)}r \end{array}$$ (18)

where *r* is, which decreases for small *t*_2_ as a consequence of the increase in AT. Inserting Eq (18) in Eq (11), the steepness of the APDR can be written as a function of RT and CV changes:

$$\begin{array}{c} \alpha \left(DI\right)=(\frac{APD_{n}^{\prime }\left(t_{2}\right)}{1-v^{\prime }\left(t_{2}\right)v^{-2}\left(t_{2}\right)r})=\frac{RT_{n}^{\prime }\left(t_{2}\right)+rv^{-2}\left(t_{2}\right)v^{\prime }\left(t_{2}\right)}{1-rv^{-2}\left(t_{2}\right)v^{\prime }\left(t_{2}\right)} \end{array}$$ (19)

Note that when CVR is not engaged, *v*′(*t*_2_) = 0, the APDR slope is simply $\alpha \left(DI\right)=APD_{n}^{\prime }\left(t_{2}\right)=RT_{n}^{\prime }\left(t_{2}\right)$, as in Eq (12).

If for a given range of PI the CVR is assumed to be a linear function of *t*_2_, i.e. $v\left(t_{2}\right)=\alpha_{V}^{0}\cdot t_{2}+c$, with $c\gg \alpha_{V}^{0}\cdot t_{2}$, then the APDR slope can be written as:

$$\begin{array}{c} \alpha =\frac{\alpha_{\text{RT}}^{0}+\left(rc^{-2}\right)\alpha_{V}^{0}}{1-\left(rc^{-2}\right)\alpha_{V}^{0}} \end{array}$$ (20)

This expression shows that *α* and the CVR slope $\alpha_{V}^{0}$ are related by an hyperbolic function similar to Eq (15), and that *α* dramatically increases within $0\leq \alpha_{V}^{0}<c^{2}/r$.

## Experimental setting and data analysis

Cardiac mapping was performed in patients undergoing cardiac surgery incorporating cardiopulmonary bypass [30]. The study was approved by the local institutional review committee at the Heart Hospital, London, UK, and all subjects gave written informed consent. Cardiopulmonary bypass was temporarily commenced to allow the surgeon to fit a multi-electrode sock enabling the recording of 240 unipolar electrograms (UEGs) over the epicardium of both ventricles. The heart was then refilled, and bypass discontinued in order to study a normally beating heart. Once baseline haemodynamics were restored, pacing impulses were delivered by a cardiac stimulator (Micropace EP Inc.), which was connected to the acquisition system composed of two amplifiers (Clearsign Lab-Sysyem Pro, Bard Electrophysiology). Duration and amplitude of the pacing pulses were set to ensure consistent capture. Unipolar electrograms were recorded with a sampling rate of 1 KHz, and band pass filtered between 0.01 Hz and 500Hz. The rib retractor was used as the reference electrode. Electrical restitution properties were studied in 8 patients (age 57 ± 16, 2 female, 6 coronary artery bypass surgery and 2 aortic valve replacement). In 6 patients, two S1–S2 pacing protocols were performed consecutively. Following a train of nine steady-state S1–S1 stimuli at 600 ms, an extra stimulus at a shorter coupled interval S1–S2 was introduced. The S1–S2 interval *t*_2_ was decremented by 50 ms steps from 550 to 350 ms; then by 20 ms steps to 330 ms; and then by 10 ms intervals until loss of ventricular capture.

Activation (AT) and repolarization (RT) times were estimated from the unipolar electrograms: AT corresponds to the time from the pacing stimulus to the minimum of the first derivative during depolarization phase, *arg*(*min*(*dV*/*dt*)), while RT corresponds to the maximum of the derivative during the repolarization phase for both positive and negative T-waves, *arg*(*max*(*dV*/*dt*)) [31]. Activation recovery interval (ARI), an established measure of APD, was calculated as *ARI*_*n*_ = *RT*_*n*_ − *AT*_*n*_ [32–34]. The diastolic interval (DI) preceding the premature beat was obtained as *DI*_*n*−1_ = *CL*_*n*−1_ − *ARI*_*x*,*med*_, where *CL*_*n*−1_ is the local CL calculated as in Eq (3) and *ARI*_*x*,*med*_ is the median ARI in beats *n* − 5: *n* − 2 (see Fig 1). This was done in order to minimize the effect of ARI dynamics on DI and to obtain a reliable DI even for very short S1-S2 coupling intervals. Maximal restitution slope, *α*, was calculated using a piecewise linear fitting strategy by performing linear regression (least square algorithm) in sliding windows 50-ms wide and recording the line with the maximal slope [29, 35]. Signal processing was performed with validated bespoke algorithms [36] as in previous studies [37, 38].

## Results from in-vivo human recording

Data from 14 restitution protocols in 8 patients were analyzed, and 1722 restitution slopes were considered after a careful selection based on signal quality and stability.

**Fig 2:**
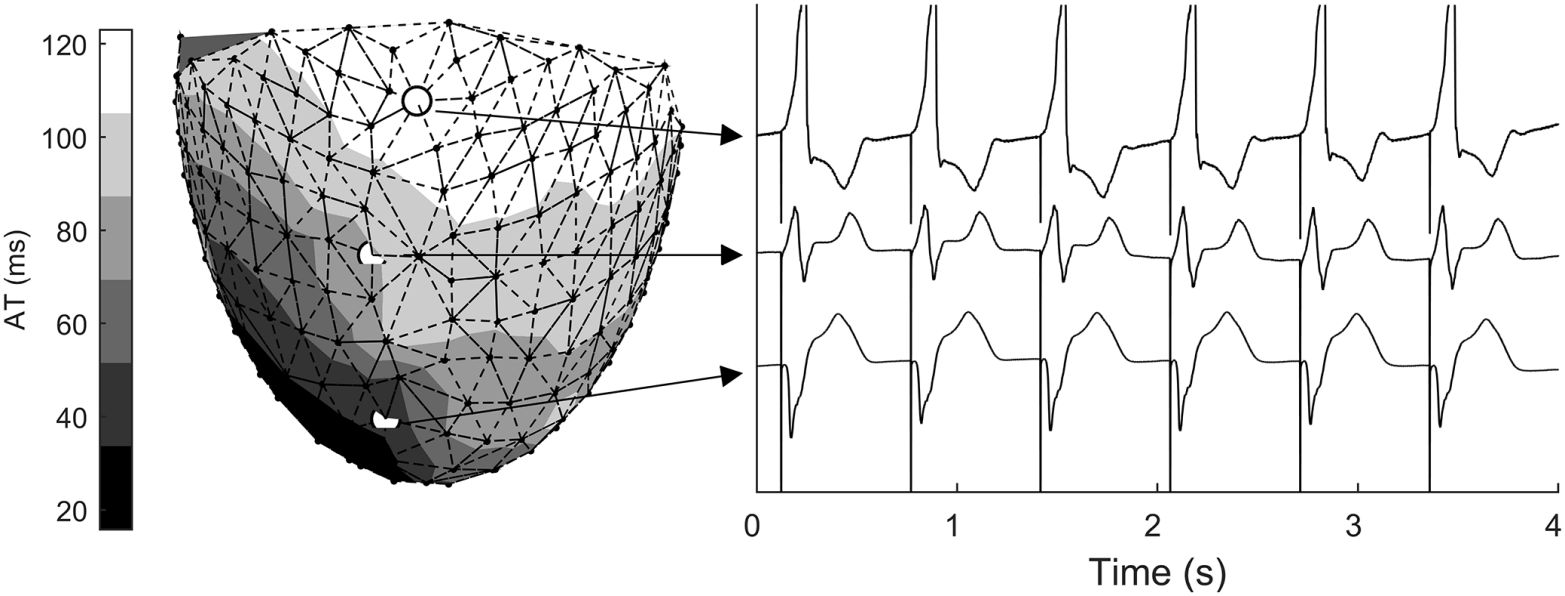
Left: Mesh of a multi-electrode sock allowing recording from 240 cardiac sites. The nodes of the mesh represent the electrodes and colors the AT. Right: Unfiltered unipolar electrograms during ventricular apical pacing. Symbols mark the position of the electrodes where signals were recorded. Signals exhibited the typical morphology associated with early (QS complex and positive T-wave), mid (RS complex and positive/biphasic T-wave), and late (R monomorphic wave and biphasic/negative T-wave) activation during ventricular pacing.

Fig 2 shows the mesh of one of the multi-electrode sock used in the study. Each node represents an electrode. Three unfiltered UEGs are also reported, exhibiting the typical morphology associated with early (QS complex and positive T-wave), mid (RS complex and positive/biphasic T-wave), and late (R monomorphic wave and biphasic/negative T-wave) activation during ventricular pacing [39]. A representative map of AT, ARI and RT during ventricular pacing is shown in Fig B in S1 File.

### Interactions of AT, APD and RT restitution properties

**Fig 3:**
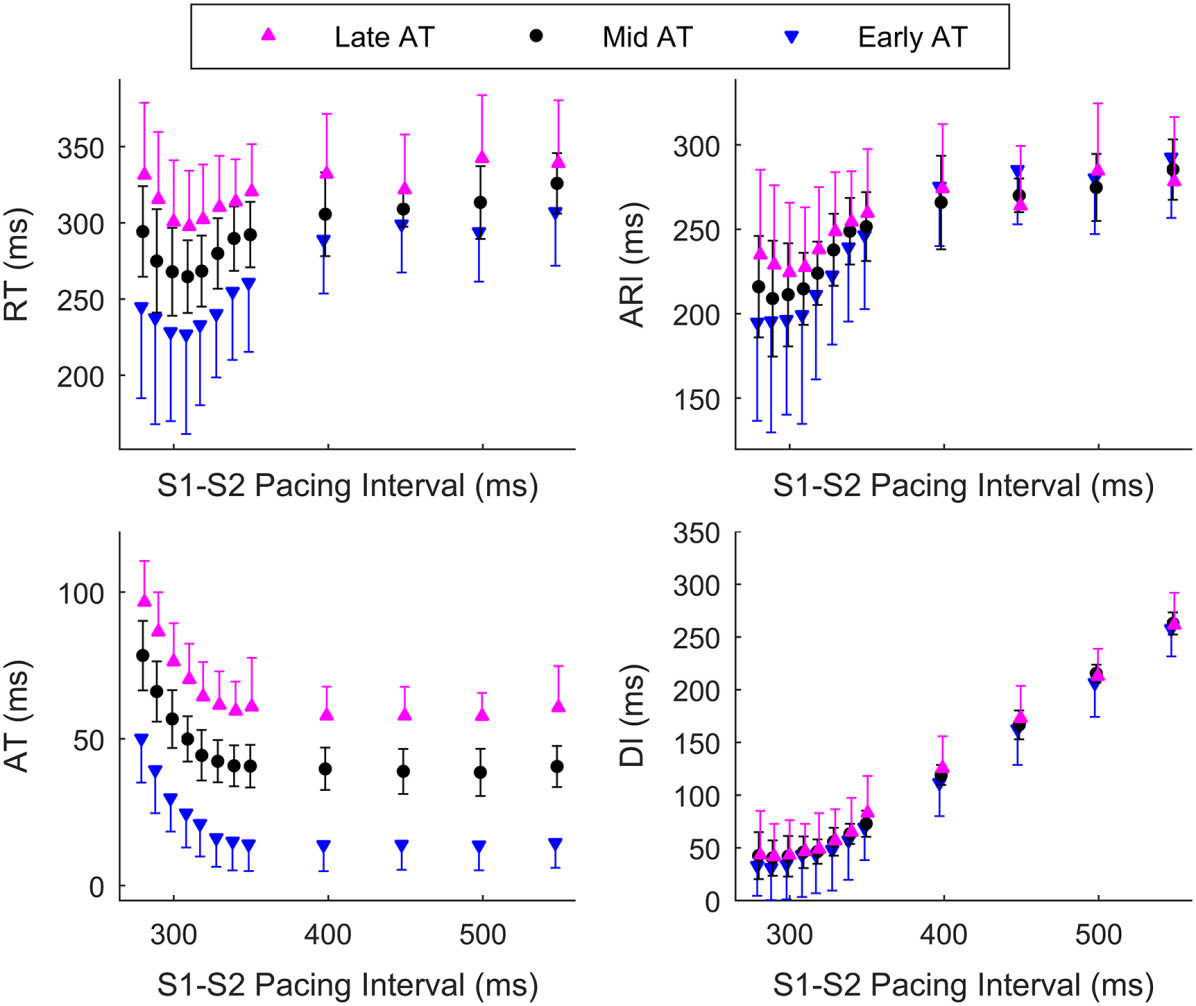
Main cardiac intervals, activation time (AT), activation-recovery interval (ARI), an established surrogate for the APD, repolarization time (RT) and diastolic interval (DI) as a function of pacing interval (PI) during an S1–S2 restitution protocol. Intervals measured in sites that activated within the first (*AT* ≤ 44.6 ms), second (44.6 < *AT* ≤ 62.7 ms) and third (*AT* > 62.7 ms) terciles are represented with different markers. Changes are commented in the text.

Fig 3 shows RT, ARI, AT and DI from a representative patient as a function of the S1–S2 interval. The cardiac intervals are divided in three groups based on the AT of the corresponding cardiac site. Sites that activated within the first (*AT* ≤ 44.6 ms), second (44.6 < *AT* ≤ 62.7 ms) and third (*AT* > 62.7 ms) terciles are represented by different markers. All relevant cardiac intervals changed with *t*_2_ and interacted to determine the restitution properties of the cardiac tissue. In this example, ARI shortened with *t*_2_ down to about 300 ms, while it flattened for lower *t*_2_. Activation time restitution was engaged for *t*_2_ < 350 ms. As a result, the dynamic of the total RT was biphasic. As expected from Eqs (5) and (10), AT restitution affected DI dynamics, which decreased with the same rate as *t*_2_ before AT restitution was engaged, i.e. in steps of Δ*t*_2_ for *t*_2_ > 300, while it flattened for *t*_2_ < 300 where AT increased. This figure also shows that restitution properties modulate the dispersion of activation and repolarization, which in this example increased for short *t*_2_ (the spread between the markers increased).

**Fig 4:**
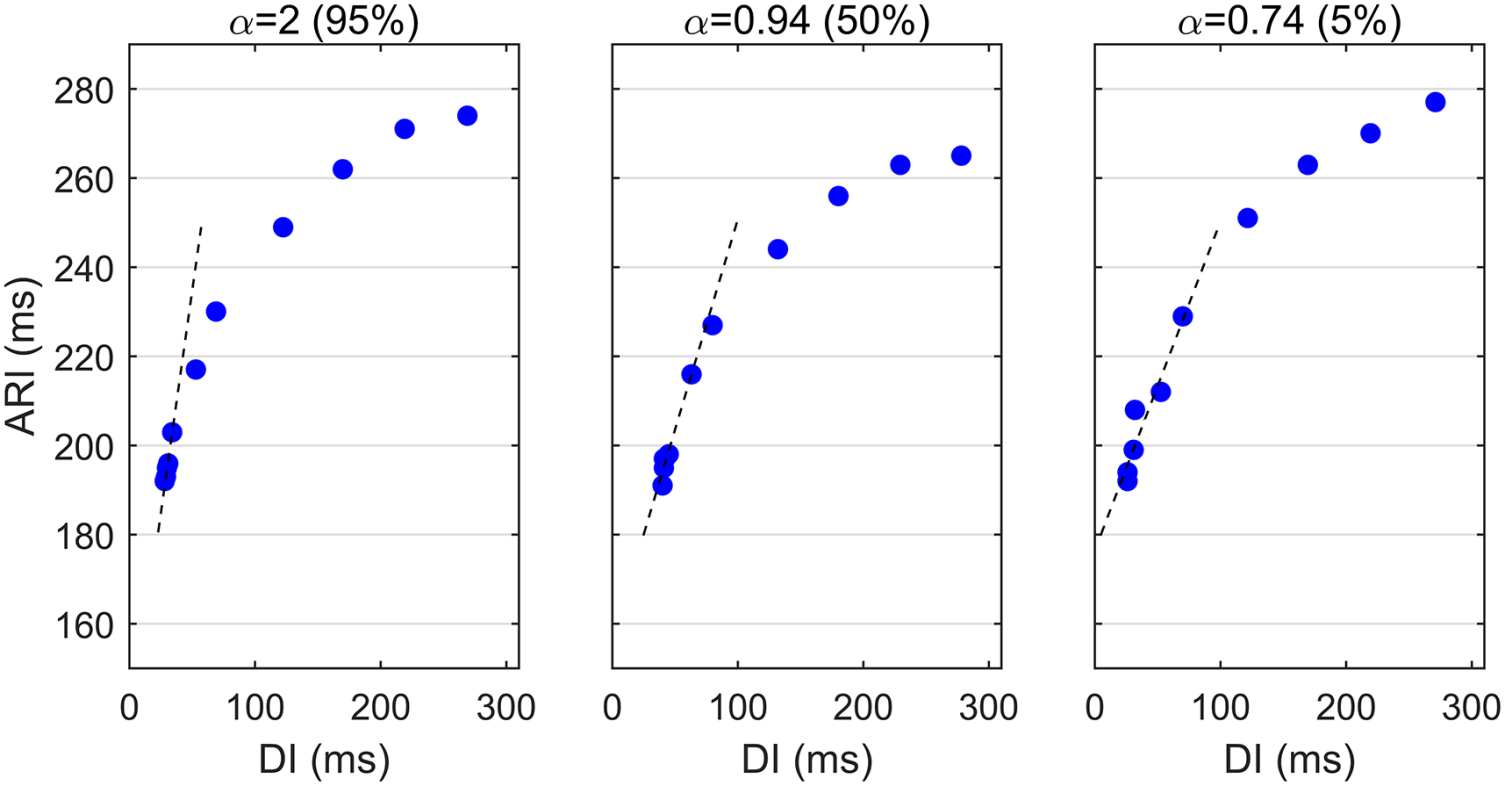
APD restitution curves in a representative patient exhibiting high, middle and low APDR slope, *α*, corresponding to 95th, 50th and 5th percentile of *α* distribution.

The dynamics shown in Fig 3 interact to promote spatial heterogeneity in the APDR slope. Fig 4 shows three APDR curves from one representative patient exhibiting low, middle and high APDR slope, which in this example spanned from 0.55 ms/ms to 3.0 ms/ms.

**Fig 5:**
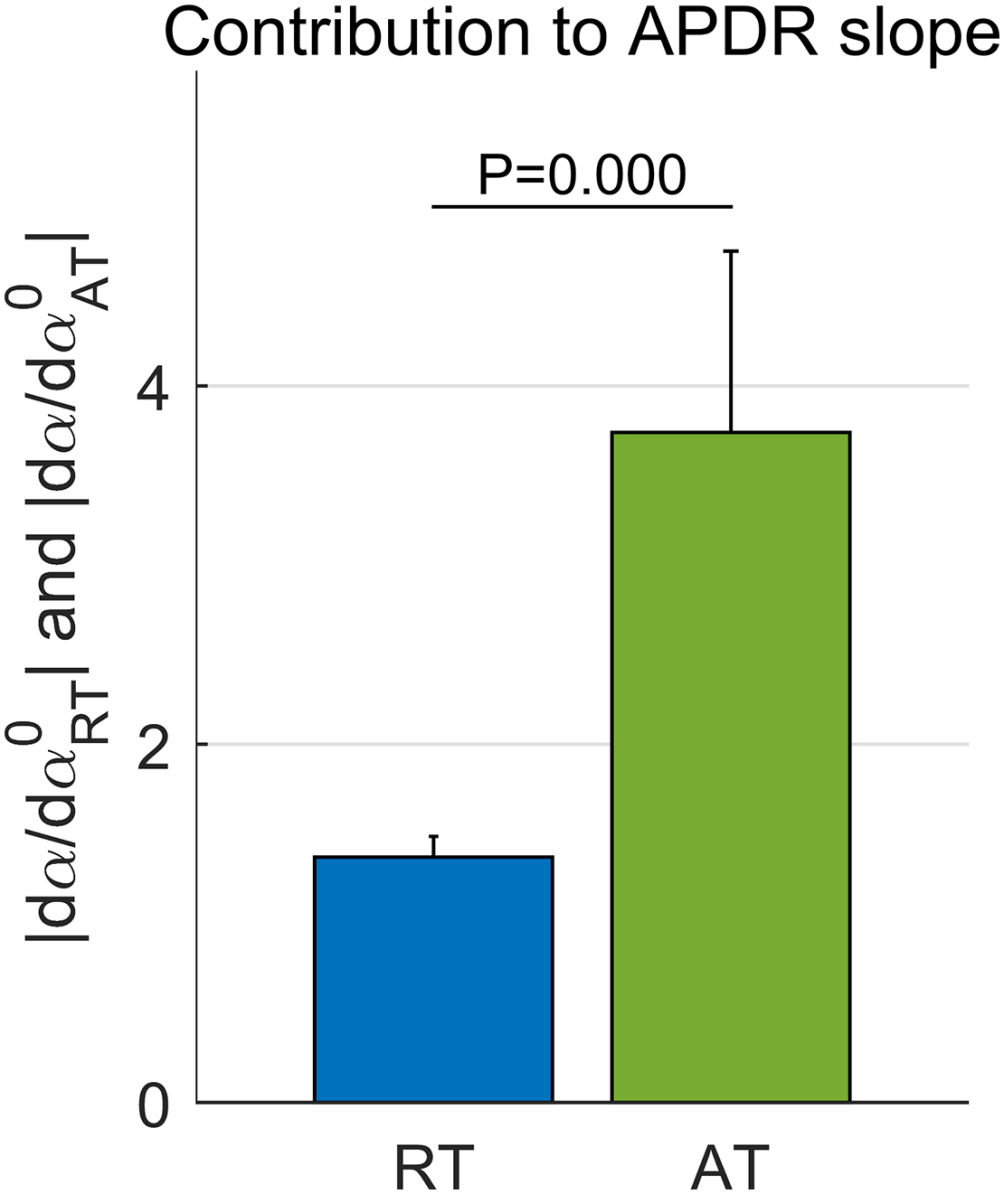
Contribution of repolarization and activation dynamics to APDR slope *α*. The contribution of AT and RT was estimated as the sensitivity of *α* to changes in $\alpha_{\text{RT}}^{\text{0}}$ (rate of change of RT with respect to S1–S2 PI) and $\alpha_{\text{AT}}^{\text{0}}$ (rate of change of AT with respect to S1–S2 PI), respectively, as in Eqs (16) and (17). Bar and lines represent the median and median absolute deviation across n = 14 restitution protocols, of median $d\alpha /d\alpha_{\text{RT}}^{0}$ and $d\alpha /d\alpha_{\text{RT}}^{0}$ estimated within each restitution protocol. Differences between $|d\alpha /d\alpha_{\text{RT}}^{0}|$ and $|d\alpha /d\alpha_{\text{AT}}^{0}|$ are statistically significant (*P* < 0.0001, Wilcoxon rank-sum test).

Fig 5 shows that the steepness of the APDR slope was almost three times more sensitive to the increase of AT than to the simultaneous reduction of RT. The relationship between changes in $\alpha_{\text{RT}}^{\text{0}}$ (or $\alpha_{\text{AT}}^{\text{0}}$) and corresponding changes in the APDR slope was estimated as $|d\alpha /d\alpha_{\text{RT}}^{\text{0}}|$ (or $|d\alpha /d\alpha_{\text{AT}}^{\text{0}}|$) as proposed in Eqs (16) and (17). On average, $|d\alpha /d\alpha_{\text{AT}}^{\text{0}}|$ was 2.7 times higher than $|d\alpha /d\alpha_{\text{RT}}^{\text{0}}|$ (*P* < 0.0001, Wilcoxon rank sum test) across all cardiac sites.

### Interdependence between APD restitution and conduction dynamics

**Fig 6:**
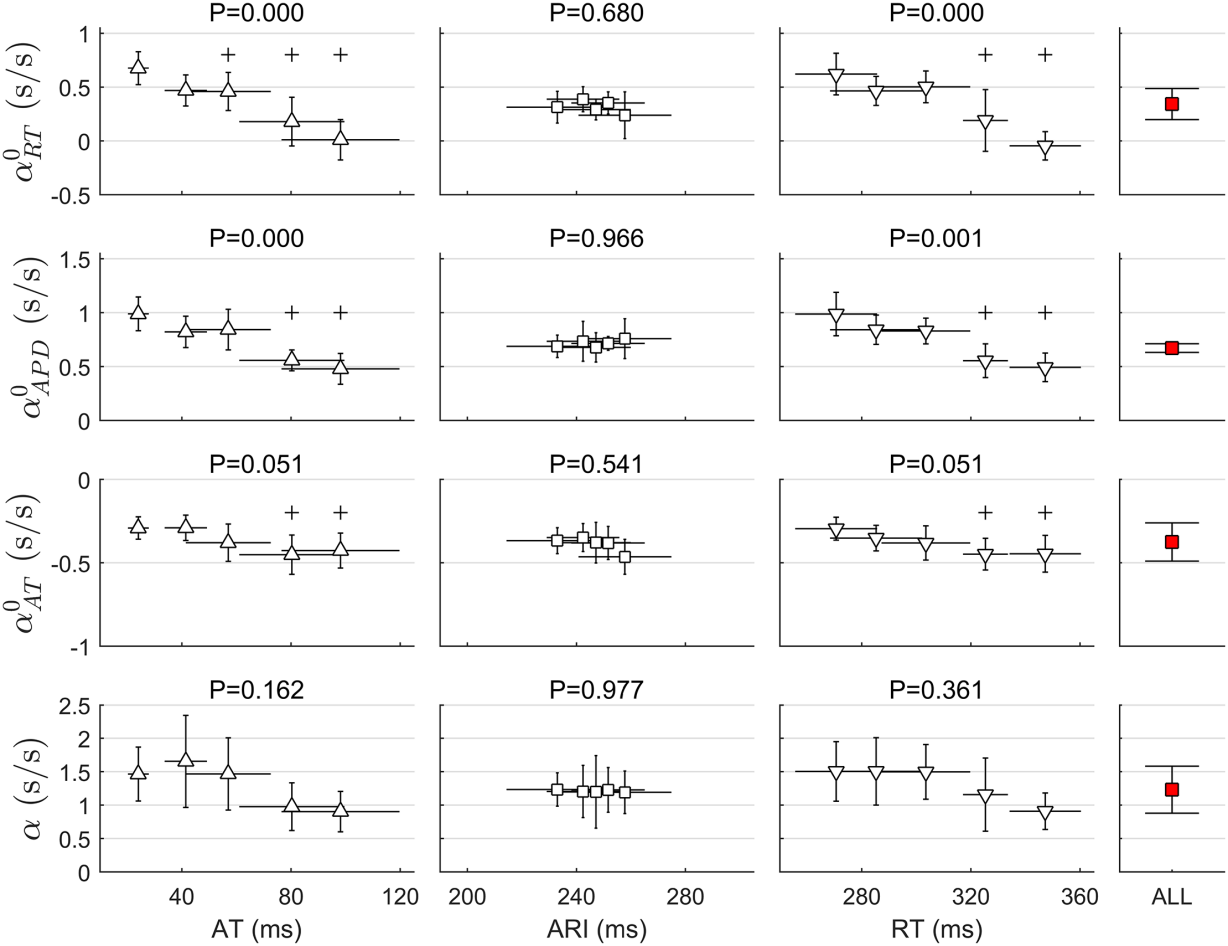
Restitution properties as a function of activation time (AT), activation-recovery interval (ARI), an established surrogate for APD, and repolarization time (RT). These intervals were divided in 5 quintiles and restitution slopes grouped accordingly. Markers and bars represent the median and median absolute deviation across restitution protocols (n = 14) of the median restitution slope within each quintile. From top to bottom: $\alpha_{\text{RT}}^{\text{0}}$, $\alpha_{\text{APD}}^{\text{0}}$ and $\alpha_{\text{AT}}^{\text{0}}$ (rate of change of RT, APD and AT with respect to S1–S2 PI) and the APDR slope *α*. Right hand column shows the median and median absolute deviation (across protocols) of median slopes within each protocol. P-value of Kruskal Wallis assessing the global difference across quintiles is reported on top of each panel. +: *P* < 0.05 (Wilcoxon rank sum test) with respect to the first quintile. For example, the upper-left panel shows that $\alpha_{\text{RT}}^{\text{0}}$ decreased with AT. Cardiac sites that activated within the last three quintiles (*AT* ≥ 46 ms ca) had a $\alpha_{\text{RT}}^{\text{0}}$ significantly lower than that of the cardiac sites that activated within the first quantile (*AT* ≤ 0.25 ms ca).

Fig 6 shows how the steepness of RT, APD and AT restitution curves changed as a function of AT, ARI and RT. Cardiac sites were grouped according to their AT, ARI and RT into five quintiles. The panels show the distribution of the median slopes calculated within each group across the 14 restitution protocols. Statistically significant pairwise differences between the slopes of a given quantile as compared to the slopes of the first quantile are marked with the symbol + (*P* < 0.05, Wilcoxon rank-sum test), while the P-value of the global differences within all groups are reported above each panel (Kruskal-Wallis test). The steepness of *RT*_*n*_(*t*_2_) and *APD*_*n*_(*t*_2_) curves, i.e. $\alpha_{\text{RT}}^{0}$ and $\alpha_{\text{APD}}^{0}$, markedly decreased along AT and RT, while they did not change for different ARI (*P* < 0.001 along AT and RT, *P* = 0.589 along ARI). The steepness of the *AT*_*n*_(*t*_2_) curve, $\alpha_{\text{AT}}^{\text{0}}$, tended to become more negative along both AT and RT, with statistically significant pairwise differences between slopes in the 4th and 5th quintiles with respect to slopes in the first quintile and almost significant global differences between groups (*P* = 0.051). As a result of the interactions between $\alpha_{\text{RT}}^{0}$ and $\alpha_{\text{AT}}^{\text{0}}$ described in Eq (15), the steepness of the APDR curve, *α*, did not change along AT or RT (bottom panels). Note that sites with different ARI did not show any difference in restitution properties, implying that the extent of ARI reduction does not depend on baseline ARI value.

The panels on the right hand side of Fig 6 show the distribution (median ± median absolute deviation across different protocols) of the median slopes calculated for each restitution protocol. These were equal to 0.26 ± 0.13 for $\alpha_{\text{RT}}^{\text{0}}$, 0.68 ± 0.07 for $\alpha_{\text{APD}}^{0}$, −0.35 ± 0.09 for $\alpha_{\text{AT}}^{\text{0}}$ and 1.15 ± 0.36 for *α*. Of note, 61% of all cardiac sites exhibited *α* > 1. As expected, the slope between APD and PI was much lower than the APDR slope, being *α* almost two times higher than $\alpha_{\text{APD}}^{\text{0}}$.

**Fig 7:**
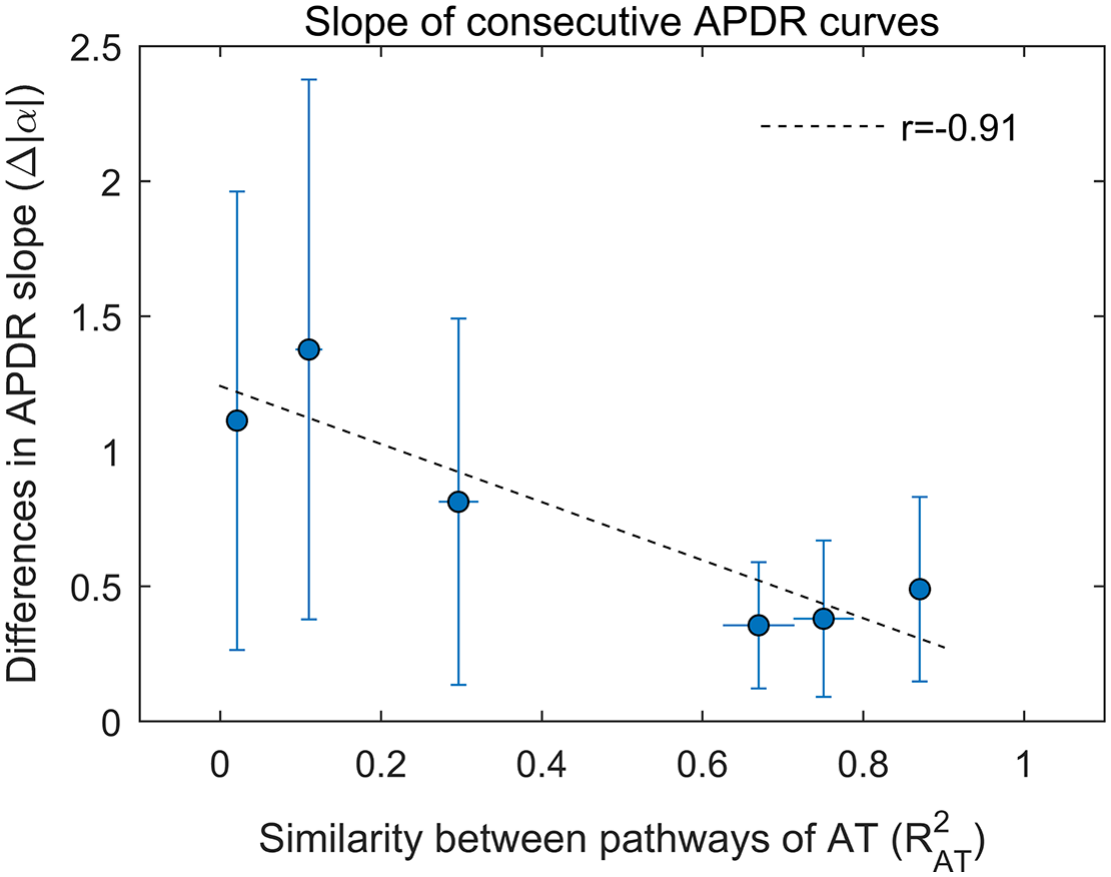
Influence of the pathway of activation on the APD restitution slope. $R_{\text{AT}}^{2}$ is the coefficients of determination between activation times (AT) for two consecutive pacing protocols. Δ|*α*| is the absolute difference between APDR slopes for the two consecutive protocols. Each marker represent each of the 6 subject for which restitution protocols were repeated twice, pacing from different sites. The higher the degree of similarity between pathways of activation (higher $R_{\text{AT}}^{2}$) the lower the difference between slopes calculated at the same cardiac sites. Markers and bars represent median and median absolute deviation of Δ|*α*| across cardiac sites. Median Δ|*α*| was linearly correlated with $R_{\text{AT}}^{2}$ with a correlation coefficient *r* = −0.91. Δ|*α*| was significantly higher for $R_{\text{AT}}^{2}<0.5$ than $R_{\text{AT}}^{2}>0.5$.

Fig 7 shows that for consecutive restitution protocols, similar APDR slopes were measured only when ventricular pacing resulted in similar pathways of activation. In six subjects, restitution protocols were repeated twice, pacing from different sites. In these subjects, the coefficient of determination $R_{\text{AT}}^{2}$, which measures the similarity between the two pathways of activation, was strongly negatively correlated with the median absolute differences between APDR slopes, Δ|*α*|, during the two different protocols (Pearson’s coefficient *r* = −0.91, Fig 7). Furthermore, Δ|*α*| was significantly higher when the two pacing protocols produced markedly different pathways of activation ($R_{\text{AT}}^{2}<0.5$) as compared to when the two pacing protocols produced similar pathways of activation ($R_{\text{AT}}^{2}>0.5$). This suggests that the spatial organization of the electrical excitation of the tissue contributes to APDR properties.

### Spatial heterogeneity of restitution properties

**Fig 8:**
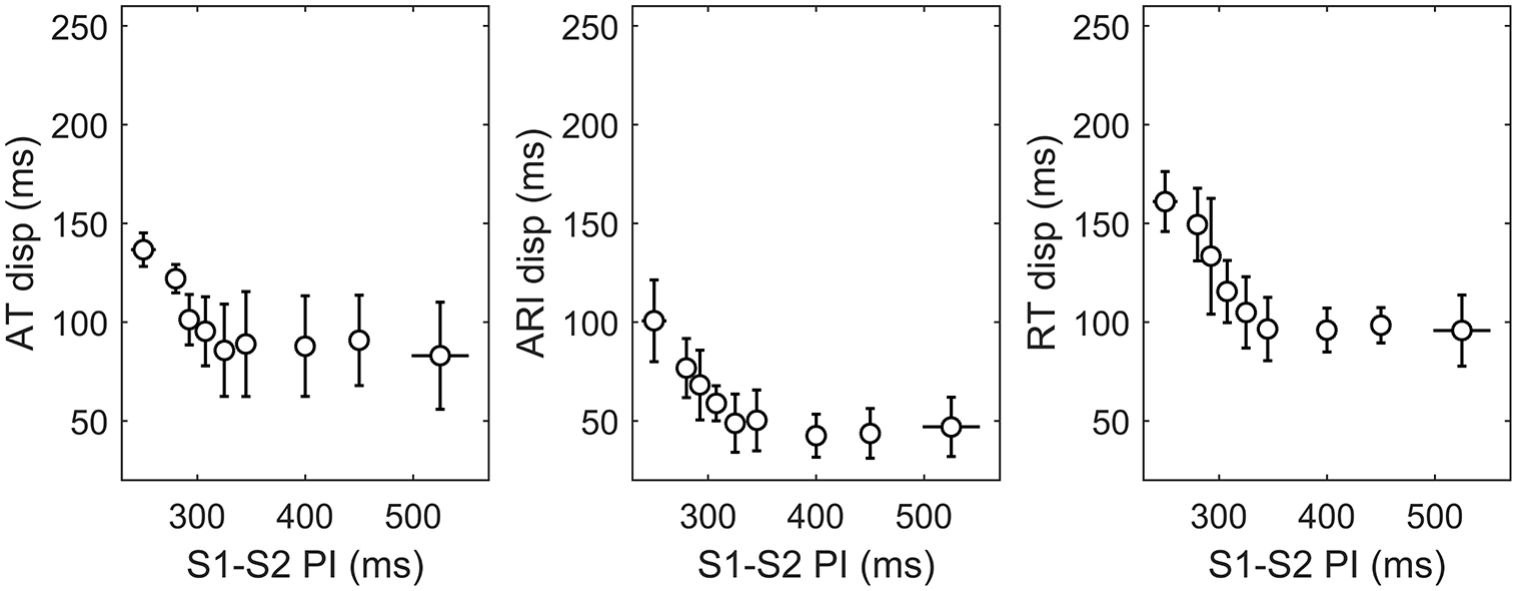
Dispersion of AT (left), ARI (middle) and RT (right) increased for short S1–S2 pacing intervals. Bars represent median and median absolute deviation of the dispersions across different protocols (n = 14). Dispersion was calculated as the difference between 95th and 5th percentile of each distribution.

Fig 8 shows that the dispersion of activation and repolarization, measured as the interval between the 5th and the 95th percentile of AT, ARI and RT, respectively, increased for short S1–S2 coupling interval *t*_2_. Dispersion of AT increased from 83 ± 27 ms for *t*_2_ > 500 ms (median ± median absolute deviation) to more than 100 ms for *t*_2_ < 300 ms. ARI dispersion increased markedly and very quickly for *t*_2_ < 320 ms, going from about 45 ms for *t*_2_ = 320 ms to more than 120 ms for *t*_2_ = 250 ms. This translated into an increase of RT dispersion from about 100 ms for *t*_2_ > 320 ms to about 200 ms for *t*_2_ = 250 ms.

**Fig 9:**
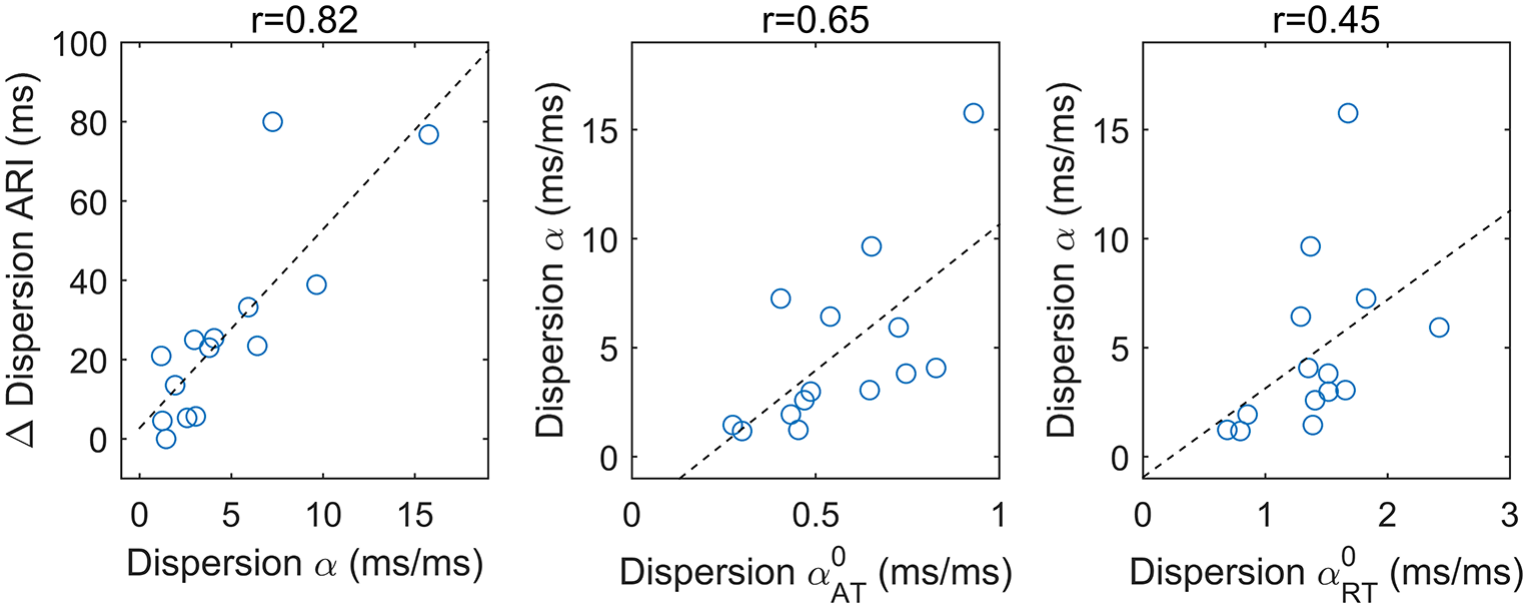
APD restitution dispersion contributed to the increase of ARI dispersion and it is due to both repolarization and activation dynamics. Each point represents a restitution protocol (n = 14). Left: APD restitution dispersion correlated with the increase in the dispersion of ARI between the largest and the shortest pacing intervals (see Fig 8-middle panel). The dispersion of APDR slope *α* correlated better with $\alpha_{\text{AT}}^{\text{0}}$ dispersion (middle), i.e. the heterogeneity of AT restitution, than with $\alpha_{\text{RT}}^{\text{0}}$ dispersion (right), i.e. the heterogeneity of the slope between RT and S1–S2 PI.

Fig 9 shows that the increase in ARI dispersion observed for short S1–S2 coupling interval was tightly correlated with the dispersion of the APDR slope (*r* = 0.82, Pearson’s correlation coefficient). The increase in ARI dispersion was measured as the difference between ARI dispersion for the two smallest and the two largest S1–S2 coupling intervals, while APDR slope dispersion was measured as the interval between the 5th and the 95th percentile in the distribution of *α*. Middle and right panels show that the dispersion in the APDR slope correlated with both $\alpha_{\text{AT}}^{\text{0}}$ and $\alpha_{\text{RT}}^{\text{0}}$ dispersions, with higher correlation coefficient for $\alpha_{\text{AT}}^{\text{0}}$ (*r* = 0.65) than for $\alpha_{\text{RT}}^{\text{0}}$ (*r* = 0.45). This demonstrates that the spatial heterogeneity of both activation and repolarization restitution properties contributed to APDR dispersion, which in turn modulated ARI and RT dispersion.

### Interpretation and validation of the mathematical formulation

**Fig 10:**
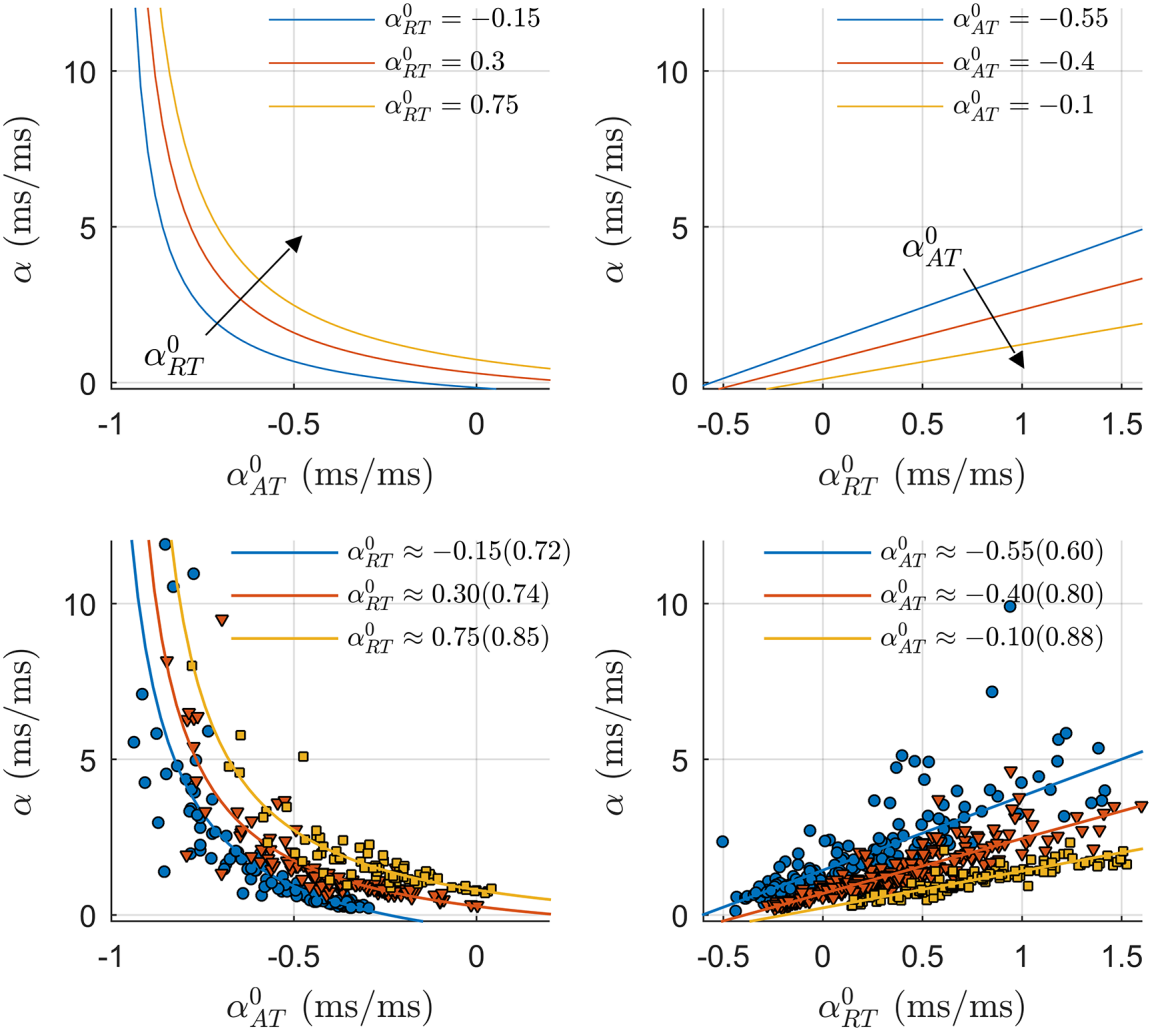
Study of the contribution of repolarization and activation dynamics to the APDR slope *α*. Upper panels show *α* as a function of $\alpha_{\text{AT}}^{\text{0}}$, the slope between AT and S1–S2 pacing interval, (left) and $\alpha_{\text{RT}}^{\text{0}}$, the slope between RT and S1–S2 pacing interval, (right) as described in the mathematical formulation in Eq (15). The non-linear hyperbolic interrelationship between *α* and $\alpha_{\text{AT}}^{\text{0}}$ implies that a small increase in AT corresponds to large increases in *α* (left). The interrelation between $\alpha_{\text{RT}}^{\text{0}}$ and *α* is linear and modulated by $\alpha_{\text{AT}}^{\text{0}}$ (right). Lower panels show that the curves reported above correctly fitted the data. Data were grouped by $\alpha_{\text{AT}}^{\text{0}}$ and $\alpha_{\text{RT}}^{\text{0}}$ to match the analytical curves described in the upper panels. Each group of data is composed of more than 70 $\left(\alpha_{\text{RT}}^{\text{0}},\alpha \right)$ and $\left(\alpha_{\text{AT}}^{\text{0}},\alpha \right)$ points. Points $\left(\alpha_{\text{AT}}^{\text{0}},\alpha \right)$ and $\left(\alpha_{\text{RT}}^{\text{0}},\alpha \right)$ were fitted by hyperbolic (left) and linear (right) functions. The coefficient of determination *R*^2^ is reported in brackets.

A graphical interpretation of the mathematical expression proposed in Eq (11) is shown in Fig A in S1 File. Fig 10 shows that the mathematical formulation derived in this paper accurately describes the data. Upper panels show APDR slope *α* as a function of $\alpha_{\text{AT}}^{\text{0}}$ (left) and $\alpha_{\text{RT}}^{\text{0}}$ (right), i.e. the changes in AT and RT with respect to S1–S2 pacing interval as represented in Eq (15). The APDR slope *α* drastically increases for more negative $\alpha_{\text{AT}}^{\text{0}}$ following a non-linear hyperbolic profile with a singularity at $\alpha_{\text{AT}}^{\text{0}}=-1$, while it increases linearly with $\alpha_{\text{RT}}^{\text{0}}$. Lower panels show that the curves reported above correctly fitted the data. Data were grouped by $\alpha_{\text{AT}}^{\text{0}}$ and $\alpha_{\text{RT}}^{\text{0}}$ to match the curves predicted by the proposed mathematical formulation and shown in the upper panels. In the left panel, cardiac sites were divided in three groups depending on $\alpha_{\text{RT}}^{\text{0}}$ (1: $\alpha_{\text{RT}}^{\text{0}}=-0.15\pm 0.05$, *n* = 130; 2: $\alpha_{\text{RT}}^{\text{0}}=0.30\pm 0.05$, *n* = 132; 3: $\alpha_{\text{RT}}^{\text{0}}=0.75\pm 0.05$, *n* = 78) and points $\left(\alpha_{\text{AT}}^{\text{0}},\alpha \right)$ followed an hyperbolic function as predicted by the proposed mathematical formulation, with coefficient of determination *R*^2^ equal to 0.72, 0.74, and 0.85, respectively. In the right panel, cardiac sites were divided into three group according to $\alpha_{\text{AT}}^{\text{0}}$ (1: $\alpha_{\text{AT}}^{\text{0}}=-0.55\pm 0.05$, *n* = 178; 2: $\alpha_{\text{AT}}^{\text{0}}=-0.40\pm 0.05$, *n* = 290; 3: $\alpha_{\text{AT}}^{\text{0}}=-0.10\pm 0.05$, *n* = 125) and points $\left(\alpha_{\text{RT}}^{\text{0}},\alpha \right)$ followed a linear regression with *R*^2^ equal to 0.60, 0.80 and 0.88, respectively.

**Fig 11:**
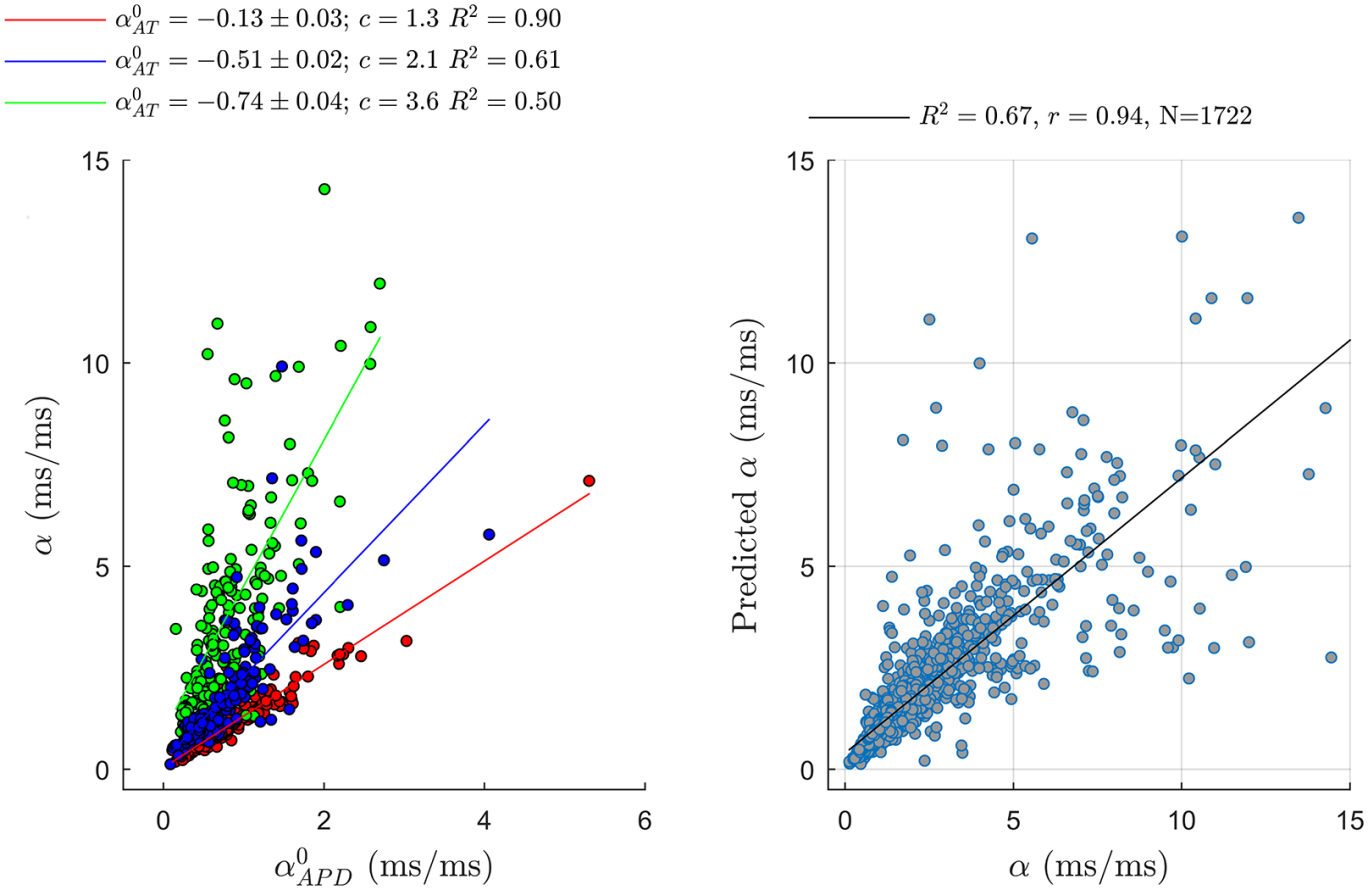
Left: Correlation between APD slope *α* and the slope between APD and S1–S2 pacing interval, $\alpha_{\text{APD}}^{\text{0}}$. Global correlation is weak (Pearson’s and Spearman’s coefficients equal to 0.44 and 0.69, respectively) and $\alpha \geq \alpha_{\text{APD}}^{\text{0}}$. The slope of the regression curve between *α* and $\alpha_{\text{APD}}^{\text{0}}$, *c*, increased with the steepness of the AT restitution $\alpha_{\text{AT}}^{\text{0}}$. *R*^2^ is the coefficient of determination of the regressions. Right: Correlation between APDR slopes predicted by the expression in Eq (15) and the APDR slope recorded in 1722 cardiac sites from 8 human hearts. Pearson’s and Spearman’s coefficients were equal to 0.82 and 0.94, respectively.

Fig 11 shows that as predicted by the proposed formulation, the extent by which the APDR slope *α* was larger than $\alpha_{\text{APD}}^{\text{0}}$ was determined by the steepness of the AT curve $\alpha_{\text{AT}}^{\text{0}}$. For shallow AT restitution curves, with $\alpha_{\text{AT}}^{\text{0}}=-0.13\pm 0.03$, *α* and $\alpha_{\text{APD}}^{\text{0}}$ were similar, with linear regression slope equal to *c* = 1.3 (*R*^2^ = 0.90). The steeper the ATR curves, the higher the ratio between *α* and $\alpha_{\text{APD}}^{\text{0}}$, with *c* = 2.1 for $\alpha_{\text{AT}}^{\text{0}}=-0.51\pm 0.02$ and *c* = 3.6 for $\alpha_{\text{AT}}^{\text{0}}=-0.74\pm 0.04$. This further demonstrates the contribution of AT dynamics to the steepness of the APDR slope. Fig 11 also shows that there was an excellent agreement between the APDR slope estimated in vivo and the slope predicted by the linear formulation in Eq (15). Considering all 1722 restitution curves, Pearson’s and Spearman’s correlation coefficients were equal to *r* = 0.82 and *r* = 0.94, respectively.

## Discussion

This paper provides an experimental and theoretical assessment of repolarization and activation time restitution properties and their interactions in the human epicardium. High density mapping of the left and right ventricles was performed during 14 restitution protocols in 8 patients, resulting in 1722 restitution curves. The experimental and theoretical results of this study demonstrate that the APDR slope, *α*, is a function of both repolarization and conduction dynamics, and it is mainly determined by the latter. The proposed mathematical formulation predicts that the APDR slope is more sensitive to AT prolongation ($\alpha_{\text{AT}}^{\text{0}}$), than to RT shortening ($\alpha_{\text{RT}}^{\text{0}}$). Accordingly, experimental results showed that the APDR slope was 2.7 times more sensitive to changes in AT than to changes in RT.

The distribution of the APDR slope throughout the epicardium was spatially heterogeneous. However, there was no correlation between the APDR slope and baseline values of ARI, AT or RT recorded at long PI, indicating that it does not change along the pathway of AT or RT and it is not a function of the local APD. APDR slope *α* > 1 was prevalent, with 61% and 28% of all cardiac sites showing *α* > 1 and *α* > 2, respectively, and median *α* = 1.15. This is in agreement with recent human studies [29, 40–44]. Due to the interactions between AT dynamics and DI, the APDR slope is steeper than the slope between APD and PI ($\alpha_{\text{APD}}^{\text{0}}$). The experimental results suggest that the APDR slope is not a sole property of local electrophysiological characteristic of the tissue, but it is also affected by the spatial organization of the electrical excitation throughout the tissue. In fact, the APDR slope recorded at the same cardiac site for two consecutive pacing protocols were similar only when ventricular pacing resulted in similar pathways of activation.

Dispersion of APDR slope was due to heterogeneity in both activation and repolarization dynamics and promoted an increase in APD dispersion at short cycle length. Global modulated dispersion [45] at shortly coupled intervals was about 150 and 250 ms for AT and RT, respectively, about 50 and 100 ms higher than at basic cycle length.

### Methodological Considerations

A number of simulation studies have investigated the possible ionic contribution to restitution properties and their link with an increased vulnerability to fatal arrhythmia [7, 17, 18, 21, 23, 46]. However, less focus has been placed on the mathematical description of the APDR slope and its interaction with conduction dynamics in tissue. The mathematical formulation presented in this paper is an analytical description of the interactions between the main cardiac intervals during changes in heart rate. In its general formulation, this mathematical description does not assume any specific dynamic for the cardiac intervals, while in its linear formulation it only requires local linearity between AT and PI, and between RT and PI, and stable conditions during pacing at basic cycle length. The tight linear correlation between estimated and predicted APDR slope (*r* = 0.82) when using the linear formulation demonstrates that these assumptions are not strong. To the best of our knowledge, and despite its simplicity, this mathematical approach to the study of restitution properties is novel.

The proposed mathematical description shows that changes in AT or RT during pacing at basic cycle length can greatly affect the APDR slope. This casts doubts on the validity of restitution analysis performed during unstable conditions, such as ventricular fibrillation [8]. The APDR slope is extremely sensitive to small changes in AT, that should therefore be measured accurately.

At short PI, the AT restitution curve becomes more and more negative while the APD flattens (e.g. Fig 3). Therefore, when comparing results from different restitution protocols it is important to compare the shape and the steepness of the APDR curve over the same range of DI, without limiting the analysis to the comparison of the maximal slope. The proposed mathematical description predicts that in case of extremely steep AT restitution ($\alpha_{\text{AT}}^{\text{0}}<-1$), the APDR curve would turn rightward for short diastolic intervals resulting in a negative APDR slope. This is sometime observed experimentally.

### Implications for arrhythmogenesis

Several studies suggest that a steep APD restitution curve can contribute to create an unstable substrate by inducing repolarization alternans [4, 7, 8], an electrical instability that may result in wave break and fibrillation [6, 38]. Recently, a steep APDR slope has also been suggested to increase the vulnerability to reentry [47, 48]. The spatial organization of restitution properties is also relevant, because a heterogeneous distribution of APDR slope may be pro-arrhythmic [42, 49]. However, some more recent in-vivo and in-silico studies have challenged the hypothesis that a simple direct causal link exists between a steep restitution curve and repolarization alternans, suggesting that other factors such as repolarization adaptation, conduction velocity restitution and electrotonic effects should be taken into account [9, 18, 40, 50]. Also, a steep restitution curve has been shown to have a poor long-term predictive value for mortality and arrhythmic events in humans [43]. Nevertheless, restitution properties remain of great interest to understand cardiac electrophysiological mechanisms, in part precisely because their role in modulating arrhythmogenesis is still undetermined.

The results of this study show that an APDR slope higher than one is prevalent in the human epicardium, suggesting that a steep APDR slope may be neither a necessary nor a sufficient condition to promote repolarization alternans. A steep APDR slope may not be a hallmark of cardiac instability, at least when it is mainly due to a steep AT restitution, a protective mechanism against conduction block and re-entry. However, APDR properties may modulate the arrhythmic risk in conjunction with other mechanisms. For example, the results of this study demonstrate that in tissue the APDR slope strongly depends on conduction dynamics. Spatial heterogeneity in AT dynamics can greatly enhance APDR dispersion, and therefore increase both AT and RT dispersion, which are key element in unidirectional block and re-entry [47, 48].

## Supporting Information

S1 File The supporting information file provides a graphical interpretation of the mathematical description of restitution properties (Fig A), and maps of activation time, repolarization time and activation-recovery intervals from a representatve patient (Fig B). (PDF)
