# Supplementary material for: Interactions between Activation and Repolarization Restitution Properties in the Intact Human Heart: In-Vivo Whole-Heart Data and Mathematical Description
Source: PLoS One. 2016 Sep 2;11(9):e0161765. doi: 10.1371/journal.pone.0161765 (PMC5010207; doi:10.1371/journal.pone.0161765)
Supplement: S1 File — (PDF) [file pone.0161765.s001.pdf]

Supporting Information Document (S1 File)

Interactions Between Activation and Repolarization Restitution Properties in the Intact Human Heart: In-vivo Whole-Heart Data and Mathematical Description

Michele Orini[1,2,*], Peter Taggart[1], Neil Srinivasan[1,2], Martin Hayward[3], Pier D. Lambiase[1,2]

1 Institute of Cardiovascular Science, University College London, London, United Kingdom

2 Barts Heart Centre, St Bartholomews Hospital, London, United Kingdom

3 The Heart Hospital, University College London Hospitals, London, United Kingdom

*** m.orini@ucl.ac.uk**

1 Supporting Information

Figure A provides a graphic interpretation of the interrelationships between activation and repolarization dynamics described by the expression proposed in (11). This diagrammatic example was produced by modifying data collected in an intact human heart and it shows two types of restitution properties.

In the first restitution curve, conduction velocity restitution is not engaged and all curves are represented by white circles. This is the case described in (12), with $AT'(t_2) = 0$ and $\alpha = \alpha^0_{\mathrm{APD}} = \alpha^0_{\mathrm{RT}}$, which in this example, as shown in both left and right panels, were equal to -0.11.

In the second case (gray markers), repolarization happens at the same time as in the previous case (not shown), but conduction velocity restitution is engaged, i.e. $AT'(t_2) \neq 0$ (see green line). As a result, $APD(t_2)$ decreases and $DI(t_2)$ flattens for short t_2. These two effects contribute to increase the steepness of the APDR slope α, reported in the right panel. The increase in AT for short PI corresponds to a negative $AT'(t_2)$ and increases $\alpha^0_{\mathrm{APD}} = \alpha^0_{\mathrm{RT}} - \alpha^0_{\mathrm{AT}}$, the slope between APD and PI, to $\alpha^0_{\mathrm{APD}} = 0.48$ (green line). This is because of the decrease in APD as described in (9). The curve $APD(t_2)$ is reported also in the right panel, for comparison with the other curves that show the APD as a function of the DI. A negative $AT'(t_2)$ also interacts with the DI and increases α through (10). The horizontal arrows shows the effect of this interaction between AT and DI changes, with a point moving from where it would be in absence of CVR to its actual site in presence of CVR. Combining these two effects, the APDR slope in presence of CVR is $\alpha = 0.83$ (red line).

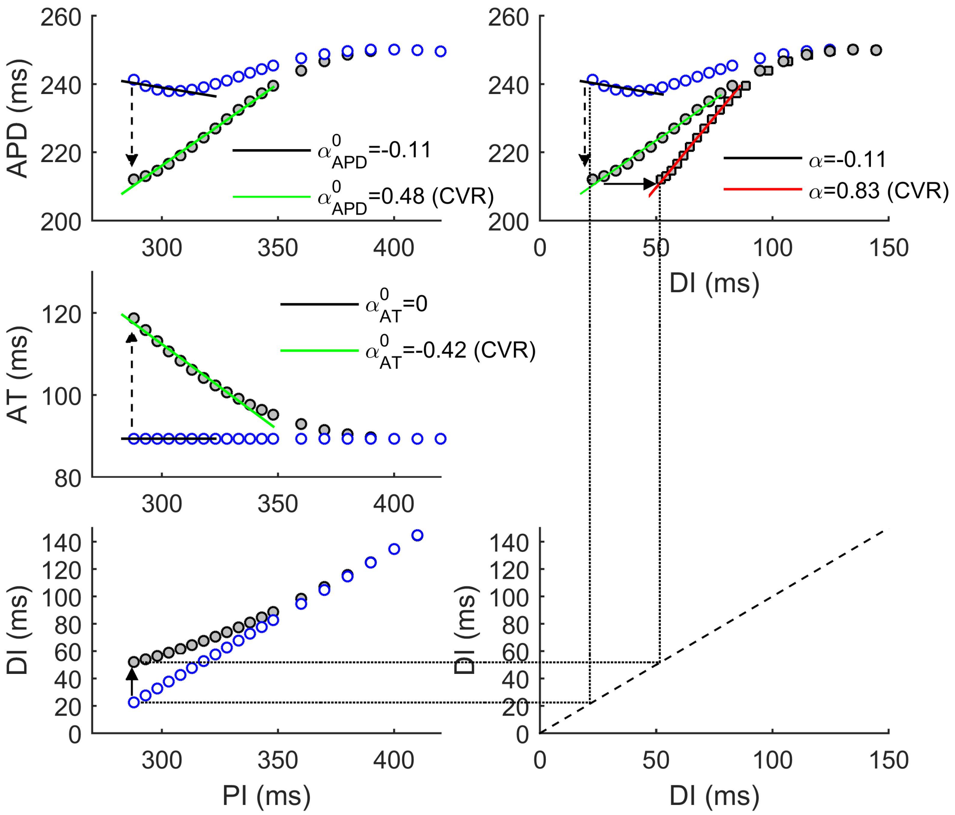

Figure A: Diagrammatic explanation of the contribution of conduction velocity dynamics to the APD restitution slope. Left: action potential duration (APD), activation time (AT) and diastolic interval (DI) as a function of pacing interval $PI = t_2$. Right: APD as a function of DI. Full and empty markers represent cases in which AT restitution is and is not engaged, respectively. When conduction velocity restitution is engaged, results are denoted with (CVR). In both examples, total repolarization dynamics are the same. AT restitution (increase of AT for short PI) has two effects: it reduces APD (dashed line arrow), and increases DI (solid line arrow). On the APD restitution slope (right panel), these two effects are reported by a dashed vertical and horizontal solid arrow, respectively. The APDR restitution slope goes from $\alpha = -0.11$ in absence of AT restitution to $\alpha = +0.83$ in presence of AT restitution.

Figure B shows a representative map of AT, ARI and RT during ventricular pacing, with electrical excitation spreading throughout the entire epicardium from the base of the left ventricle in about 100 ms and total RT following a similar pattern.

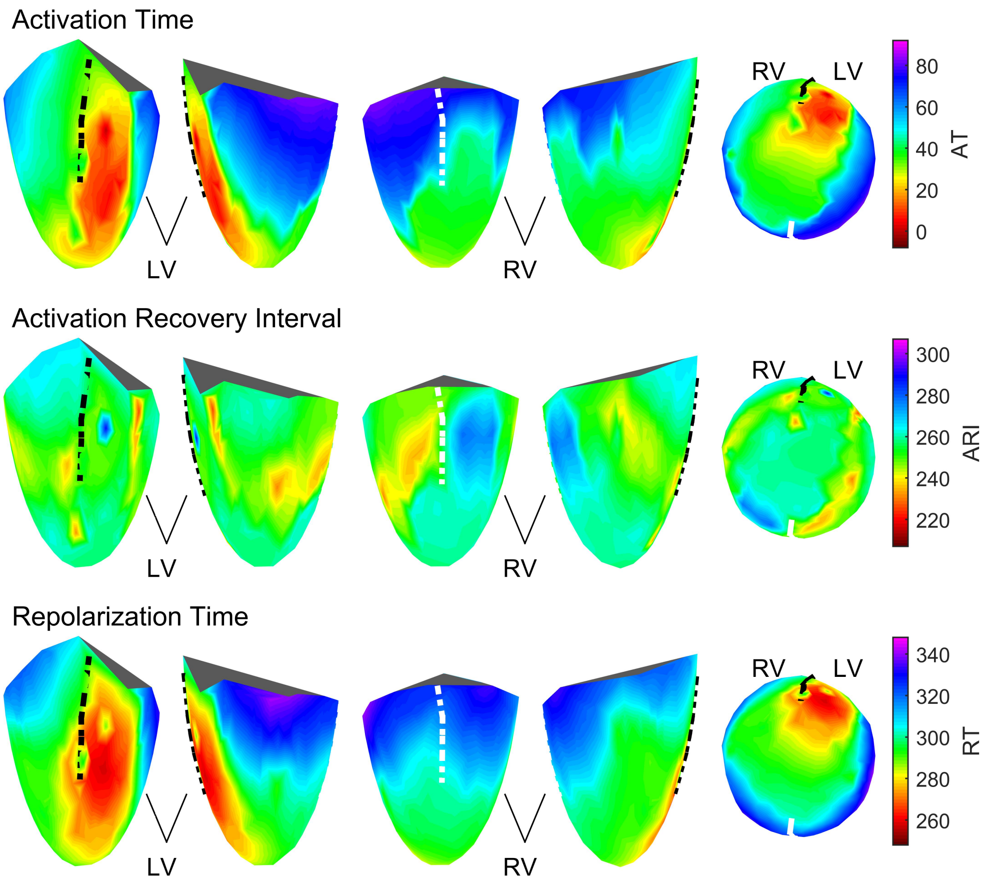

Figure B: From top to bottom : Maps of activation time (AT), activation-recovery interval (ARI) and repolarization time (RT). Different columns represent the same map for different views. Dashed black line represents the left anterior descending (LAD) artery, separating the left and right ventricles.
